# Antimicrobial Brazilian Propolis (EPP-AF) Containing Biocellulose Membranes as Promising Biomaterial for Skin Wound Healing

**DOI:** 10.1155/2013/703024

**Published:** 2013-06-09

**Authors:** Hernane da Silva Barud, Adalberto Miguel de Araújo Júnior, Sybele Saska, Letícia Boldrin Mestieri, Juliana Alvares Duarte Bonini Campos, Rubens Moreno de Freitas, Nathália Ursoli Ferreira, Andresa Piacezzi Nascimento, Felipe Galeti Miguel, Mirela Mara de Oliveira Lima Leite Vaz, Edna Aparecida Barizon, Franciane Marquele-Oliveira, Ana Maria Minarelli Gaspar, Sidney José Lima Ribeiro, Andresa Aparecida Berretta

**Affiliations:** ^1^Instituto de Química, Universidade Estadual Paulista (UNESP) CP 355, 14800-900 Araraquara, SP, Brazil; ^2^Faculdade de Odontologia, Universidade Estadual Paulista (UNESP) Rua Humaitá 1680, 14801-903 Araraquara, SP, Brazil; ^3^Laboratório de Pesquisa, Desenvolvimento e Inovação (P, D and I)-Apis Flora Industrial e Comercial Ltda. Rua Triunfo 945, 14020-670 Ribeirão Preto, SP, Brazil; ^4^Departamento de Ciências Farmacêuticas, Faculdade de Ciências Farmacêuticas de Ribeirão Preto, Universidade de São Paulo, Avenida do Café, s/n, 14040-903 Ribeirão Preto, SP, Brazil

## Abstract

Among remarkable discoveries concerning propolis, such as antifungal, antiviral, and antioxidant activities, its anti-inflammatory, and mainly its antibacterial, properties deserve special attention when skin wound healing is concerned. Based on this and knowing the distinctive performance of bacterial (BC) membranes on wound healing, in this work it is proposed to demonstrate the potent antimicrobial activity and wound healing properties of a novel propolis containing biocellulose membrane. The obtained propolis/BC membrane was able to adsorb propolis not only on the surface, but also in its interstices demonstrated by scanning electron microscopy, X-ray diffraction, Fourier transform infrared (FT-IR) spectroscopy, and thermogravidimetric assays. Additionally, the polyphenolic compounds determination and the prominent antibacterial activity in the membrane are demonstrated to be dose dependent, supporting the possibility of obtaining propolis/BC membranes at the desired concentrations, taking into consideration its application and its skin residence time. Finally, it could be suggested that propolis/BC membrane may favor tissue repair in less time and more effectively in contaminated wounds.

## 1. Introduction

The comprehensive therapeutic applicability of propolis has been demonstrated for centuries, if not for millennia, and nowadays there are innumerous scientific reports elucidating the action mechanism claimed for the compounds of this balsamic resin. Among remarkable discoveries, such as antifungal [1, 2], antiviral [3] and antioxidant activities [4–6], its anti-inflammatory [7], and mainly its antibacterial [8–10], properties deserve special attention when skin wound healing is concerned.

The worldwide occurrence of skin wounds, such as burn injuries, remains high despite efforts to reduce injury incidence through public awareness campaigns and improvements in living conditions. Severe burn injuries, as well as other forms of stress and trauma, trigger a hypermetabolic response requiring aggressive resuscitation, nutrition, excision, grafting, and pharmacotherapeutic regimen [11]. On the other hand, infection is still one major problem that may lead to death. It has been estimated that 75% of deaths following burn injuries are related to infection [12, 13].

Additionally, it has been reported that a challenge faced in the treatment of skin wounds and in burn injuries is the dressing. Some properties for dressing have been addressed: (i) low cost, (ii) safe, (iii) relatively painless, (iv) prevention of infection, (v) promotion of fast wound healing, (vi) low number of dressing changes during healing, and so forth [13].

In this regard, the last decades have witnessed an increased interest in the use of biomaterials, for example, biopolymers, in healthcare products, especially in dressing for wounds, a fact that is predominantly associated to the renewable nature, biocompatibility and biodegradability of these materials [14]. Among several biopolymers of interest, biocellulose or bacterial cellulose (BC) produced by the *Gluconacetobacter *genus is extremely pure and allows obtaining highly swollen membranes, with around 99% water on the culture medium surface [15]. In addition, distinctive tridimensional and branched nano- and microfibrillar structure is formed leading to considerable interest not only as dressing for wound healing, but also as substitutes of natural skin [16] and as drug delivery systems [14, 17]. Moreover, it has been demonstrated that BC present mechanical properties, such as tensile strength and extensibility, similar to human skin and it allows the growth, spreading, and migration of human keratinocytes [18]. 

Meanwhile, BC membrane itself has no antimicrobial activity to prevent wound infection [19]. In the last years, antimicrobial silver nanoparticles containing BC membranes have been developed using different routes and reducing agents [19–21].

Based on the exposed advantages provided using biocellulose and following our interest in the elucidation of propolis activity, especially regarding its properties on skin [5, 6, 8], in this work we reported preparation, comprehensive characterization, and efficacy of novel Brazilian propolis containing biocellulose membrane. Therefore, the main purpose was to demonstrate the antimicrobial activity and the wound healing properties of this new device material.

## 2. Material and Methods 

### 2.1. Materials

Green propolis standardized extract (EPP-AF) was kindly provided by Apis Flora Co. (Ribeirão Preto, São Paulo, Brazil) (patent PI 0405483-0, Revista de Propriedade Industrial No. 1778 of 01/02/2005). For quantitative analysis caffeic, *p*-coumaric and *trans*-cinnamic acids (Sigma-Aldrich, São Paulo, Brazil), artepillin C (Wako Pure Chemical Industries Co., Osaka, Japan), and aromaden-drin-4′-O-methyl ether (previously isolated and identified as described by Souza et al. [4] and kindly donated by the authors) were used. Methanol HPLC-grade was obtained from J. T. Baker and water was treated in a Milli-Q water purification system. All other chemicals were of reagent grade and were used without further purification. Mueller Hinton agar (Difco, Detroit, MI, USA) was used for the antibacterial assay.

### 2.2. Methods

#### 2.2.1. Bacterial Cellulose/Propolis Membranes Preparation

BC membranes were obtained from cultivation of the *Gluconacetobacter hansenii* ATCC 23769. Culture media were established for 120 h at 28°C in trays of 30 × 50 cm, containing the sterile media composed of glucose 50 g·L^−1^, yeast extract 4 g·L^−1^, anhydrous disodium phosphate 2 g·L^−1^, heptahydrated magnesium sulphate 0.8 g·L^−1^, and ethanol 20 g·L^−1^. After 120 h, hydrated BC pellicles (5 mm thick) were obtained. These membranes were several times washed in water, 2% aqueous NaOH at 70°C in order to remove bacteria, and water until neutral pH. Next, the BC membranes were immersed in ethanol for 24 h with continuous exchanges performed in order to ensure substitution of water for ethanol. Ethanol swollen BC membrane was used to prepare the BC/propolis samples.

In the preparation of BC/propolis membranes, firstly alcoholic propolis solutions were prepared at 1.2%, 2.4%, and 3.6% (w/v considering the dried matter in the propolis extract) employing 11% of Green Propolis Standardized Extract EPP-AF (w/v). Ethanol swollen BC membranes were immersed for 24 h in these solutions. BC/propolis membranes were dried at 40°C for 24 h and were set in nylon molds. Samples were named BC/propolis A, BC/propolis B, and BC/propolis C, respectively.

#### 2.2.2. Physical-Chemical Characterization

*Scanning Electron Microscopy (SEM)*. Scanning electron microscopy FEG-SEM (JEOL JMF-6700F—Field Emission, Scanning Electron Microscopy) was used to observe the surface topography of all samples. All specimens were placed in copper supports, covered with a tick carbon layer.

*X-Ray Diffraction*. The X-ray diffractograms were obtained using a Siemens Kristalloflex diffractometer (Siemens, Knoxville, TN, USA) with a nickel filter and radiation CuK_*α*_ between 2*θ* angle from 4 to 70°, counting time of 2 s, and glass sample holder.

*Fourier Transform Infrared (FT-IR) Spectroscopy.* FT-IR spectra were obtained with a Perkin-Elmer spectrometer, model 2000. Samples were milled and mixed with dried KBr in known proportions and pressed into pellets. 

*Thermal Analysis.*The thermogravimetric assays (TGAs) were carried out using a SDT 2960 equipment from TA Instruments. Samples were heated at a constant rate of 10°C min^−1^ from 25°C to 450°C, under a nitrogen flow of 70 mmL min^−1^. 

*Chemical Characterization*. Analyses were conducted using a Shimadzu (Kyoto, Japan) liquid chromatograph equipped with a CBM-20A controller, a LC-20AT quaternary pump, a SPD-M 20A diode-array detector, and Shimadzu LC solution software, version 1.21 SP1. A Shimadzu Shim-Pack CLC-ODS column (4.6 mm × 250 mm, particle diameter of 5 *μ*m, and pore diameter of 100 Å) was used. The mobile phase consisted of methanol (B), and of a solution of water-formic acid (0.1% v/v), pH 2.7 (A). The method consisted of a linear gradient of 20–95% of B over a period of 77 min at a flow rate of 0.8 g mL^−1^. The injection volume was 10 *μ*L. The column oven was set at 40°C. Detection was set at 275 nm [8, 22].

Working solutions were prepared daily in methanol in the following concentration ranges: caffeic acid 1.06–16.96 *μ*g mL^−1^; *p*-coumaric acid 5.04–80.64 *μ*g mL^−1^; *trans*-cinnamic acid 0.40–6.40 *μ*g mL^−1^; aromadendrin-4′-O-methyl ether 2.0–32.0 *μ*g mL^−1^ and artepillin C 10.06–160.96 *μ*g mL^−1^. 

The samples of BC/propolis were weighed (25 mg) on an analytical balance and transferred into 10 mL volumetric flasks. Next, the volume was completed with methanol and it was sonicated for 30 min for maximum release of propolis. The samples were filtered through a 0.45 *μ*m filter before analysis.

#### 2.2.3. Antibacterial Assay

The disk diffusion method (Clinical and Laboratory Standards Institute (CLSI)) [23] was employed in this study, with some modifications, to evaluate the antibacterial activity of the BC/propolis against *Staphylococcus aureus* ATCC 25923, *Staphylococcus aureus* ATCC 43300, and *Staphylococcus epidermidis *ATCC 14990.

The bacterial suspension was prepared in a sterile 0.85% physiological solution, with turbidity equivalent to a 0.5 McFarland standard (approximately 10^8^ CFU mL^−1^). A sterile cotton swab was used to seed the suspension on the surface of Mueller Hinton agar contained in a plate (90 × 15 mm).

Membranes were cut in disks (diameter 5.5 mm) and applied to the agar surface. Next, 5 *μ*L of sterile 0.85% physiological solution were put on the surface of each biomembrane. Biomembranes without propolis submitted to the same procedure of the BC/propolis were used to control the experiment. The plates were incubated at 35°C aerobically for 18 h. After the incubation period, the diameters of the zones of inhibition were measured using a ruler. The experiments were replicated three times for each microorganism.

#### 2.2.4. *In Vivo* Experiment

**Figure 1:**
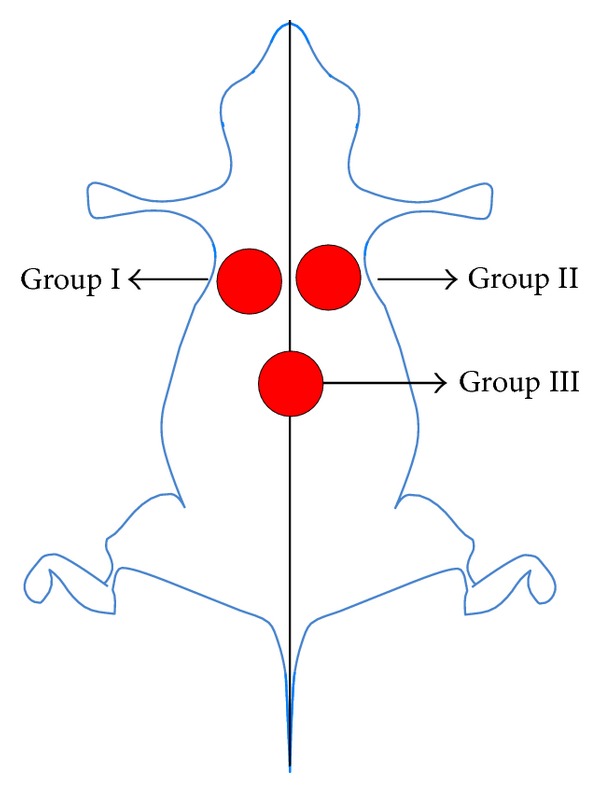
Identification of the wounds created surgically for the respective treatment groups.

This experiment was approved by the Animal Experimentation Ethics Committee of Araraquara University (UNIARA), Araraquara, SP, Brazil. Twenty-four male rats (Rattus Norvegicus Holtzman), weighing approximately 250 g, were used in this study. General anesthesia was induced using intramuscular injections of ketamine hydrochloride (25 mg kg^−1^) and xylazine hydrochloride (5 mg kg^−1^). All surgical procedures were performed under strict aseptic protocol. After shaving and preparation of the dorsal region, on each animal were made three incisions of 6 mm in diameter with a circular scalpel and a distance of approximately 2 cm among them (Figure 1) [8].

On the left side, group I (G1), propolis-free membrane was put on the wound (positive control) and on the right side, group II (G2), BC/propolis membrane (B) was put on the wound. In group III (G3), median region, the wound was untreated (negative control). Subsequently, the animals were accommodated in cages properly isolated and were monitored daily, with appropriate conditions of food, water, and hygiene. In the immediate postoperative period, all animals received an oral administration of salicylic acid (120–300 mg kg^−1^, single dose), as recommended by the Canadian Council on Animal Care (CCAC).

The animals (*n* = 6, per group) were euthanized after 3, 7, 15, and 30 days postoperatively. Firstly, the animals were anesthetized again following the protocol described previously, and after the specimens were removed, the animals received deepening anesthetic. The specimens were fixed in 10% buffered formaldehyde solution and processed according to, histological routine for light microscopy. The specimens were then cut in 6 *μ*m sections in the longitudinal direction of the skin and stained with hematoxylin-eosin (H&E). Next, they were analyzed and photographed under a microscope (Jenaval-Zeiss) coupled to a digital camera (Leica DFC425). The parameters analyzed were descriptive and examined by a single researcher who was blinded to analysis of the groups. The parameters analyzed were, (1) inflammatory reaction, (2) angiogenesis process, and (3) quality of tissue repair. 

*Macroscopic Assessment*. The wounds were analyzed macroscopically after periods of 3, 7, and 15 days postoperatively. The 30-day period was excluded from the analysis because all the wounds of the three study groups had already been fully repaired. Evaluation of the wounds was performed by measuring lesion in the longitudinal and transverse direction (mm) using a caliper rule, during the preeuthanasia procedure. For statistical analysis, the measurements, longitudinal and transverse, of each wound were added and divided by two for obtaining a repair average value.

#### 2.2.5. Statistical Analysis

The data of antibacterial assay were submitted to the nonparametric Kruskal-Wallis test. Statistical significance was established at *P* < 0.01. Statistical analysis of data was performed using the software Graph Pad Prism 4. For the *in vivo* study, statistical analysis was performed using SPSS 20.0 software. Sphericity and normality tests were performed previously. A two-way ANOVA was applied to macroscopic assessment. Statistical analysis in relation to intensity of inflammatory reaction was measured by score (score 0 to 4: no reaction = 0, very slight reaction = 1, mild reaction = 2, and moderate reaction = 3; marked reaction = 4), following the standards of ASTM F981-04 [24]. In this analysis, twenty histological cuts were analyzed for each specimen in the respective periods. The ANOVA test was carried out to evaluate measured scores to intensity of inflammatory reaction, and Tukey's test was used as posttest for statistical significance. Statistical significance was established at *P* < 0.05.

## 3. Results and Discussion

### 3.1. Physical-Chemical Characterization

#### 3.1.1. Scanning Electron Microscopy (SEM)

**Figure 2:**
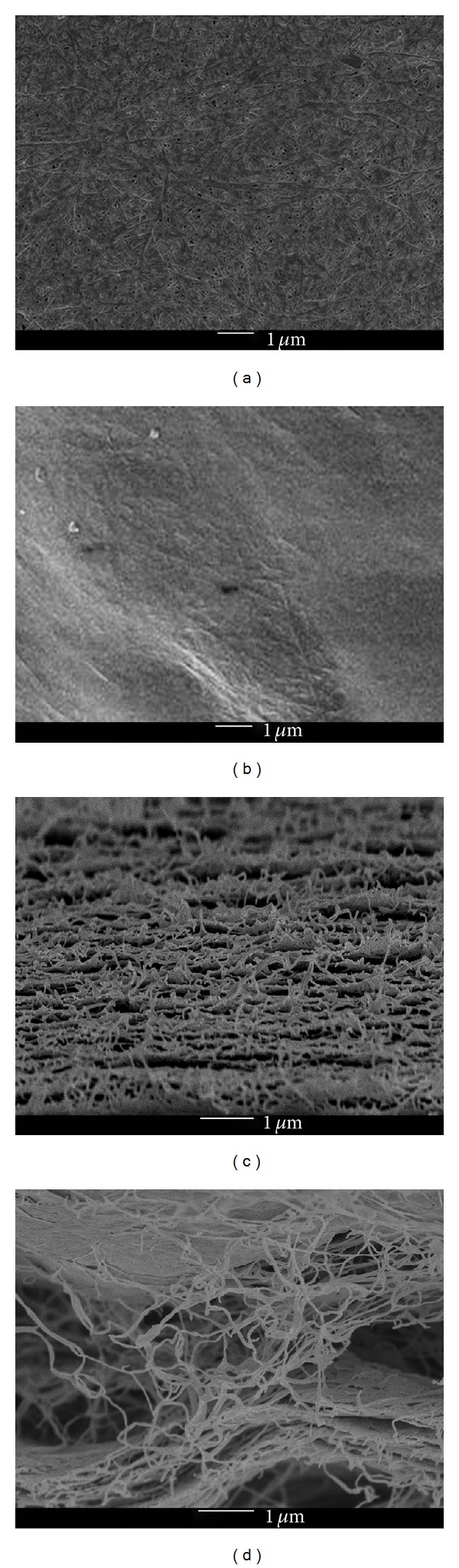
Representative SEM images of (a) BC membrane and (b) BC/propolis B surface images and (c) pristine BC membrane and (d) BC/propolis B cross-sections images.

BC is a semitransparent paper-like material, and the final BC/propolis was obtained as a flexible and macroscopic homogeneous membrane. BC/propolis membranes presented amber colorations in a dose-dependent way. SEM measurements were performed for some representative samples, including BC membrane and BC/propolis B, Figure 2.

BC membrane (Figure 2(a)) clearly presents a compact structure composed of long fibers of hundreds of microns with nanometer thickness [16].  Figure 2(b) reveals that BC microfibril is swollen and homogeneously covered by propolis extract solution. Cross-section images, Figures 2(c) and 2(d), confirm that propolis is present not only in the surface of BC membrane but also inside the cellulose chains. 

#### 3.1.2. X-Ray Diffraction

**Figure 3:**
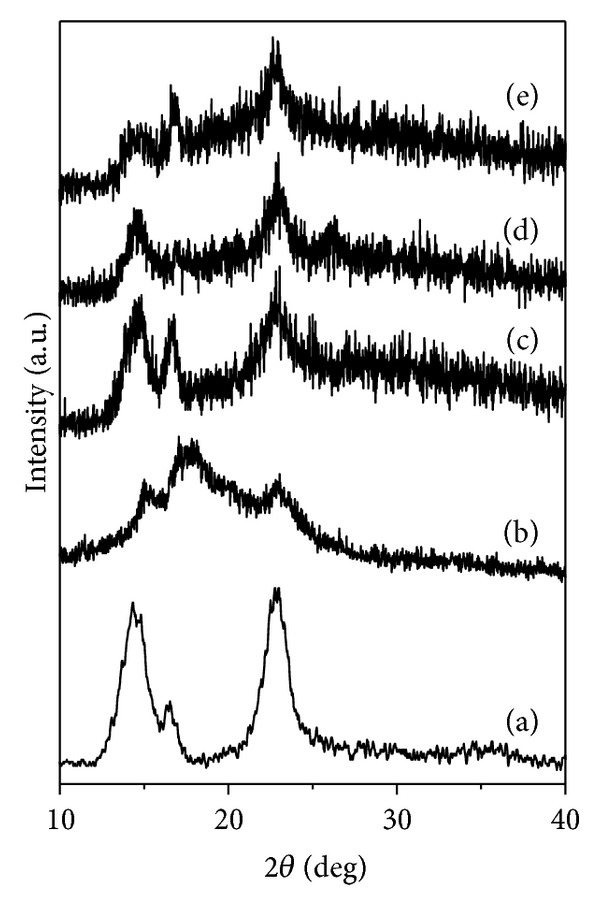
XRD diffractogram of (a) BC membrane, (b) dried propolis, (c) BC/propolis A, (d) BC/propolis B, and (e) BC/propolis C.

Figure 3 shows the results of X-ray diffraction for all samples. The BC membrane presented two diffraction angles, 15 and 22.5° degrees (Figure 3(a)). Each peak has a contribution of diffractions corresponding to I*α* and I*β* phases, characteristics of native cellulose type I [25]. 

Dried propolis extract also showed an amorphous structure, and three broad peaks in 15°, 17°, and 23° could be marked, corroborating with previous results for propolis [22].

All BC/propolis membranes showed considerable change on X-ray profile when compared to BC membrane. A gradual decrease in crystallinity is observed with increasing of propolis contents, inferring that the propolis extract solutions is not only on BC surface, but it is found in the interstices and between the crystal planes of the polymer, making it more amorphous. 

#### 3.1.3. Fourier Transforms Infrared (FT-IR) Spectroscopy

**Figure 4:**
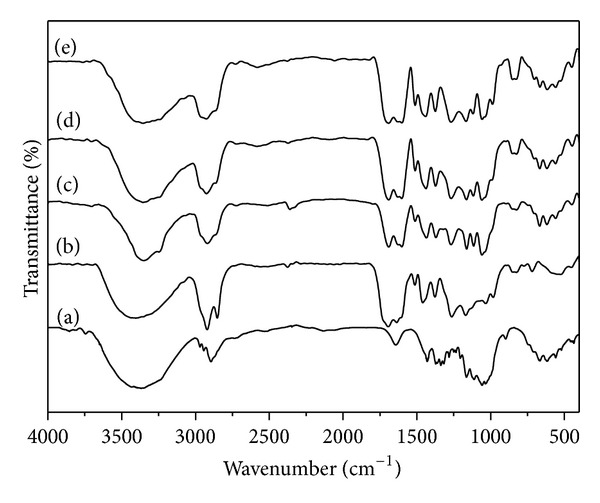
FT-IR spectra of: (a) BC membrane, (b) Dried propolis, (c) BC/propolis A, (d) BC/propolis B and (e) BC/propolis C.

Figure 4 shows the results of FT-IR spectroscopy for BC, propolis, and all BC/propolis membranes. 

BC (Figure 4(a)) shows bands in the 400–700 cm^−1^ range characteristic of the OH bending, *β*-glucosidic linkages between the glucose units at ~896 cm^−1,^ and C–O symmetric stretching of primary alcohol and C–O–C antisymmetric bridge stretching at 1040 and 1168 cm^−1^, respectively. The C–H deformation (CH_3_ or O–H in plane bending) is seen at 1340 cm^−1^, and the band centered at 1400 cm^−1^ is related to CH_2_ bending and OH in plane bending. Other bands are related to H–O–H bending of adsorbed water (at 1650 cm^−1^), CH stretching of CH_2_ and CH_3_ groups (at 2900 cm^−1^), and OH stretching (broad band at 3500 cm^−1^) [26].

Some bands that characterize propolis in the IR spectrum are absorptions related to the presence of C=C double bond (*ν*max 1641 cm^−1^), carboxyl (*ν*max 1703 cm^−1^), and hydroxyl (*ν*max 3487 cm^−1^) groups, with concern to organic compounds.

Spectra concerning BC/propolis-based membranes can be considered as the sum of the bands present in BC membrane and in propolis; in other words, there are observed contributions relating to characteristic vibrations both of the BC membrane [27] and propolis [22], showing, therefore, the formation a composite material. Some peaks were slightly shifted due to the formation of intermolecular interactions, like hydrogen bonding, between propolis component and cellulose.

Broadening was observed for the band around 3500 cm^−1^ with the increase in the propolis relative content, suggesting the formation of hydrogen bonds between cellulose and propolis components such as flavonoids. 

#### 3.1.4. Thermogravidimetric Assay

**Figure 5:**
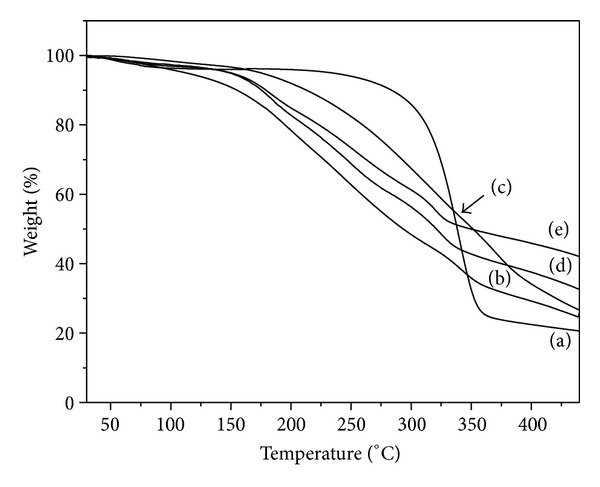
TG curves of (a) BC membrane, (b) dried propolis, (c) BC/propolis A, (d) BC/propolis B, and (e) BC/propolis C.

Thermogravimetry (TG) curves for BC membrane and BC/propolis membranes are shown in Figure 5. BC membrane displayed a typical weight-loss degradation profile (Figure 5(a)) with two main separate degradation steps: the first step (45–150°C) can be attributed to the cellulose dehydration while the second step, with large mass loss, (250–350°C) is related to processes of cellulose degradation as depolymerization and subsequent decomposition of the glycosidic units followed by the formation of carbonaceous residues [27, 28].

Propolis presented a single weight loss profile with large mass loss (65%) in the temperature range of 150–450°C, which can be attributed to simultaneous events, including condensation of groups (OH–), carbonic bond breaks, and subsequent degradation of organic compounds [22].

BC-propolis samples showed a thermal profile similar to the one obtained for dried propolis. BC/propolis membranes presented a dehydration process starting around 50°C suggesting that after being topically applied they are able to keep the wound surface hydrated, avoiding loss of electrolytes [29].

A continuous weight loss in the temperature range of 90–450°C is observed for all BC-propolis samples, Figures 5(c) and 5(d). These events include condensation of groups (OH–), carbonic bond breaks, and subsequent degradation of propolis organic compounds, and they also include BC membrane degradation [22, 27, 29].

An increase in the final residue content with the increase in the relative propolis content was observed confirming that the propolis mass is dose-dependent on the initial propolis solutions (1.2, 2.4, and 3.6% w/v) used in BC/propolis membrane preparation. 

Determination of dry matter of propolis in the samples of BC/propolis A, B, or C was performed by an indirect way using HPLC results and comparing with the standard values found in propolis standardized extract used. The results showed that BC/propolis A, B, and C possess, respectively, 43.67 ± 10.62%, 56.78 ± 15.22%, and 66.59 ± 13.45% w/w of propolis, fact observed because of the large superficial area of never-dried BC membrane in contact for a long time with propolis solutions (large ability to adsorb large mass) and the open porous present during this kind of manufacture process [30].

#### 3.1.5. Chemical Characterization

The quantification of some standards present in BC/propolis membranes was performed by HPLC. The employed method was fully validated as reported by Rocha et al. [31]. The results for BC/propolis biomembranes A, B, and C, and the propolis standardized extract (EPP-AF) used, may be observed in Table 1. As expected, the quantified compounds were dose dependent, therefore, assuming that there is a greater amount of active compounds inserted in biomembrane C, followed by biomembrane B and biomembrane A.

 Analysis of BC/propolis composition comparing with propolis extract used, after dry matter normalization, shows that the flavonoid aromadendrin was the component better adsorbed in BC, probably because of intermediary polarity of this component. On the other hand, artepillin C, the major constituent of EPP-AF propolis extract [8], showed relatively low adsorption in BC (75.34%, 72.59%, and 84.69%, BC/propolis A, B, and C, resp.) probably due to lower polarity of this structure when compared with caffeic acid derivatives and because of intrinsic properties of BC. Caffeic acid, *p*-coumaric and cinnamic acids were well adsorbed.

### 3.2. Antibacterial Assay

Propolis-free membranes (negative control) showed no antibacterial activity. On the other hand, the different membranes presented antibacterial activity (Table 2) with the BC/propolis membrane C being the most effective (*P* < 0.01).

The results presented in Table 2 are lower than the results presented by de Rezende et al. [32] for propolis ethanolic extract, especially for *S. aureus* ATCC 25.923 (15 mm), *S. aureus* ATCC 6538 (17 mm), and *S. aureus* ATCC 29213 (11 mm), using the same methodology. However, it is important to consider that the diffusion of the substances in this type of methodology completely affects the results found. It is possible that the strong interaction previously demonstrated for propolis and BC decreases the liberation and diffusion of the propolis compounds in the medium, and, therefore, a lower inhibition zone is observed.

The samples were effective against all staphylococci tested, including *S. aureus *ATCC 43300, a methicillin-resistant *S. aureus *(MRSA). These strains are responsible for difficult-to-treat infections in humans, as they exhibit multidrug resistance, such as penicillins, cephalosporins, and carbapenems.

*S. aureus *and coagulase-negative staphylococci (like *S. epidermidis*) are between the most frequently isolated bacteria from burn wounds. Furthermore, in burn wound microbial colonization there is an initial predominance of these bacteria and other gram-positive cocci over gram-negative bacilli [12]. These results suggest that the BC/propolis may be an effective material to be used for infected wounds, which would work not only as an excellent dressing, but also as an antibacterial one. Additionally, the results obtained in the physical-chemical characterization and the antibacterial activity of the BC/propolis membrane show that these parameters are dose dependent, supporting an interesting possibility, that is, obtaining propolis membranes at the desired concentrations, taking into consideration its application and its skin residence time.

### 3.3. Histological Analysis

BC membrane, in contrast to other synthetic membranes, is highly resistant to chemical corrosion, biocompatible, porous, and also has good tensile strength [33], unique properties that make it a polymer with great potential to be explored by biotechnology and other areas of biomedical science, mainly employing the BC as potential carrier for drug delivery systems. 

In the last decades, BC membranes have been used as a temporary dressing of skin in burns and other conditions such as chronic ulcers and grafts [34–38]. In addition, *in vivo* studies have demonstrated that BC membranes are biocompatible and can be used as scaffold for tissue engineering [39–41]. 

The studies were performed only for the BC/propolis B, once this membrane presented good results in the previous antibacterial studies. The inflammatory reactions presented in the studied groups were shown in Table 3. 

**Figure 6:**
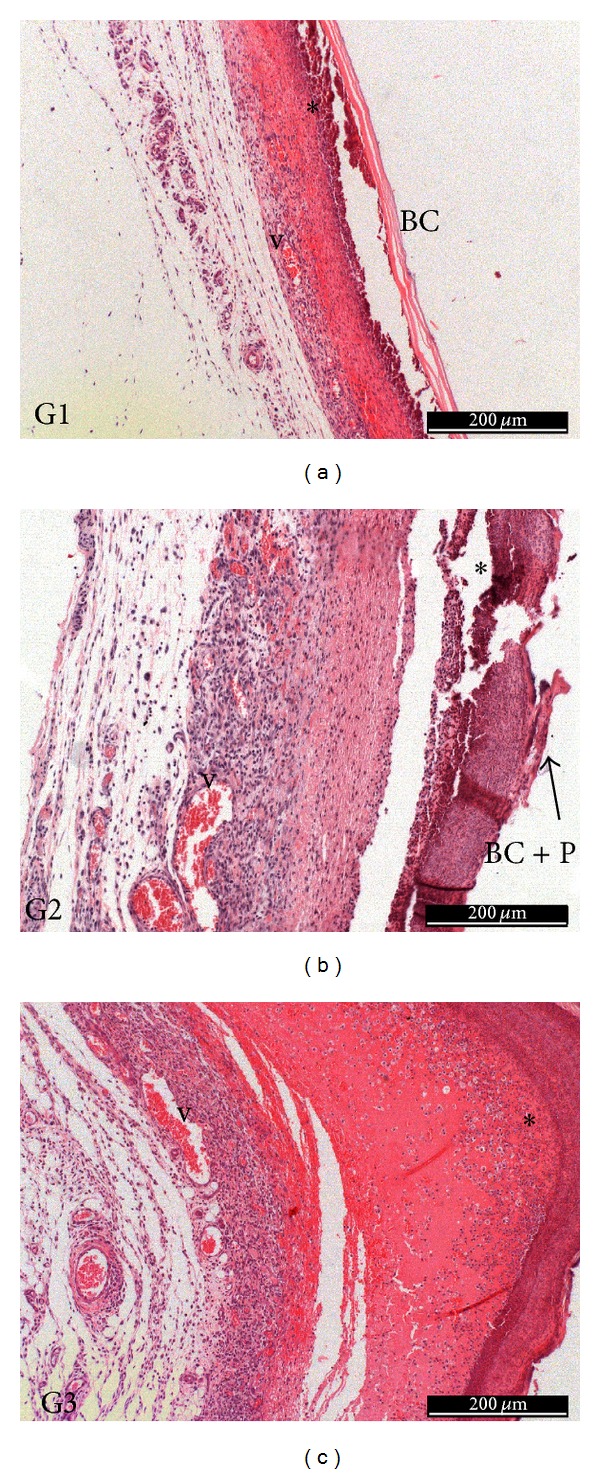
3 days of treatment—G1: necrotic tissue (*), BC membrane (BC), and presence of moderate inflammatory infiltrate and numerous blood vessels (v); G2: necrotic tissue (*), BC/propolis membrane (BC), presence of moderate inflammatory infiltrate and numerous blood vessels (v); G3: necrotic tissue (*), presence of moderate inflammatory infiltrate and numerous blood vessels (v).

**Figure 7:**
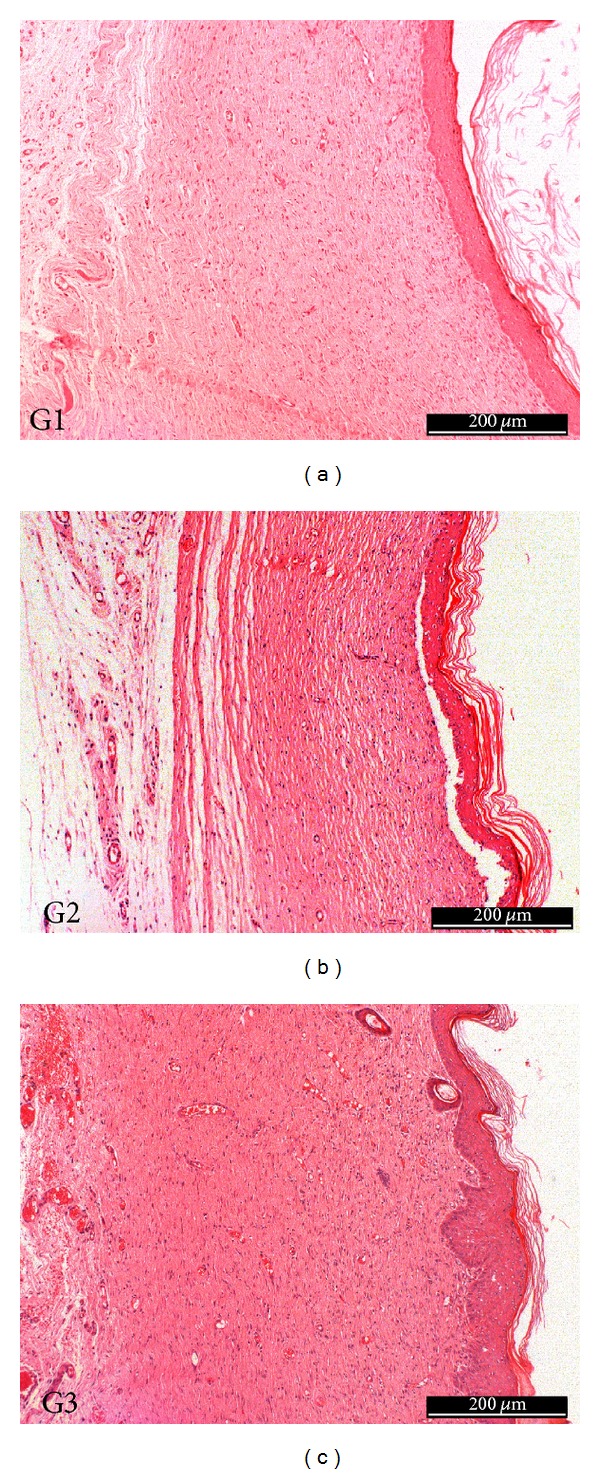
30 days of treatment—repair tissue with normal characteristics in all three groups.

The histological analyses of the wounds healing are shown in Figures 6 and 7. At day 3, the wound bed appeared in reorganization; necrotic tissue (fibrin-leukocytes crust), mainly in the control group, whose tissue can also be observed under respective membranes, BC and BC/propolis, also appeared. Beneath this tissue, it is observed that formation of granulation tissue abundant constituted of few disorganized collagen fibers with large fibroblastic activity (loose connective tissue) and presence of numerous blood vessels in stasis (Figure 6).

After 7 days, a thin layer of epidermal reepithelialization can be observed, and loose connective tissue is still shown in repair process in all tested groups, but collagen fibers were denser and organized in relation to earlier period and presence of numerous blood vessels, mainly in the treated groups with membranes as dressing, G1 and G2. Moreover, the repair of the epithelium also appeared in a more advanced stage in these respective groups, compared to the control group (G3), presenting continuity in the epithelium surface. It was observed the presence of necrotic tissue in all groups. At day 15, repair of the epithelium (keratinized stratified squamous epithelium), absence of a discontinuity and presence of keratohyalin granules in the three groups (stratum corneum) were observed. The repaired fibrous conjunctive tissue presented normal characteristics, where were observed a great number of thick-regular collagen fibers, decreasing the amount of blood vessels. At day 30 (Figure 7), the histological characteristics were similar to the period of 15 days with great deposition of collagen in this tissue and accentuated compression of the newly formed capillaries. 

Propolis is a known anti-inflammatory agent [42, 43], and then the reduction of inflammatory process was expected in G2 group. Some authors attribute this activity to the important biomarker of green propolis, artepillin C [44]. The anti-inflammatory effect of artepillin C was observed, and the authors have claimed that this property is mediated, at least in part, by prostaglandin E2 and nitric oxide inhibition through NF-*κ*B modulation [44]. 

The results obtained are important and satisfactory and revealed that BC and BC/propolis membranes were biocompatible and promoted, in earlier periods, a better tissue repair. Also, BC/propolis presented a delivery system, since preliminary results of propolis delivery from BC/propolis was obtained through *p*-coumaric acid, isossakuranetin, and artepillin C release evaluation using Franz Cell assay. Results with 24 hours of evaluation demonstrated that p-coumaric acid was released in a maximum of 60.21%, isosakuranetin 53.89%, and artepillin C 37.26%. However, it is important to consider that epithelial tissue was completely reconstituted with the application of a thermoreversible gel with propolis standardized extract (EPP-AF) within 3 days after injury [8]. In this case, the gel promotes hydration of wound for a longer time favoring the healing process; thus, with wound hydration and covering, there is no crust formation, factors that favor a shorter wound resolution. A hypothesis to solve the problem of dryness of the BC membranes would be including in the treatment protocol the rehydration of membrane; this way, we could assess if there would be improvement and decrease of the time of wound healing. 

**Figure 8:**
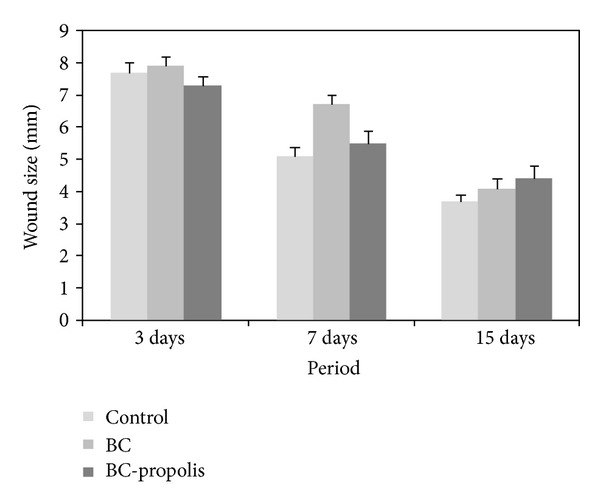
Macroscopic results obtained with tissue repair with BC, BC/propolis, and without treatment (control) (*P* > 0.05).

So, the results of the macroscopic analysis revealed repair values around 7.6 mm, 5.8 mm, and 4 mm after 3, 7, and 15 days postoperatively (Figure 8). Macroscopically, the repair of wounds showed no statistically significant differences (*P* > 0.05) among the treatments employed with the BC and BC/propolis membranes and the control group (no dressing) during the period studied; the results were confirmed by histological analysis in relation to layer of epidermal reepithelialization, and these results also suggest that the described hypothesis above may have relevance. Therefore, the type of treatment does not affect the repair time for uncontaminated wounds.

## 4. Conclusion

The present study suggests the potential applicability of propolis containing biocellulose membranes for treating wounds. The obtained BC/propolis was able to adsorb propolis not only on the surface, but also in its interstices demonstrated by SEM, X-ray diffraction, FT-IR spectroscopy, and thermogravidimetric assays. Additionally, the caffeic acid derivatives and flavonoid determination and the prominent antibacterial activity in the BC/propolis demonstrated to be dose dependent, supporting an interesting possibility, the obtention of propolis based membranes at the desired concentrations, taking into consideration its application and its skin residence time. Finally, it could be suggested that BC/propolis membrane may favor tissue repair in less time and more effectively in contaminated wounds.

## Figures and Tables

**Table 1:** Chemical composition of propolis standardized extract (EPP-AF) and bacterial cellulose/propolis A, B, and C (mg/g). Values are mean ± SD obtained from analyses in triplicate.

Standards Researched	Average ± SD			
	EPP-AF	A	B	C
Caffeic acid	0.345 ± 0.011	1.30 ± 0.074	1.65 ± 0.113	1.95 ± 0.130
*p*-coumaric acid	1.712 ± 0.069	6.10 ± 0.389	7.86 ± 0.195	9.25 ± 0.266
*Trans*-cinnamic acid	0.167 ± 0.006	0.66 ± 0.075	0.88 ± 0.031	0.99 ± 0.060
Aromadendrin	0.808 ± 0.024	4.50 ± 0.274	6.01 ± 0.089	6.61 ± 0.306
Artepillin C	6.621 ± 0.306	19.78 ± 3.70	24.81 ± 5.04	33.95 ± 2.24

SD: standard deviation.

**Table 2:** Mean diameter (in mm) of the zones of inhibition provided by biomembranes A, B, and C. Values are mean ± SD (*n* = 3).

Bacterium	Bacterial cellulose/propolis		
	A	B	C
BC without propolis	*R*	*R*	*R*
*Staphylococcus aureus* ATCC 25923	8 ± 0.58	9 ± 0.00	10 ± 0.00
*Staphylococcus aureus* ATCC 43300	7 ± 0.00	8 ± 0.00	9 ± 0.00
*Staphylococcus epidermidis* ATCC 14990	7 ± 0.58	8 ± 0.00	9 ± 0.00

SD: standard deviation. R: resistant.

**Table 3:** The levels of the inflammation reaction to the respective studied groups in comparison with the analyzed periods.

Period		Group	
	G1	G2	G3
3	+++*	+++*	+++*
7	−	++*	−
15	−	−	−
30	−	−	−

G1: propolis-free membrane; G2: BC/propolis B membrane and G3: negative control. Score: − no reaction, + very slight, ++ mild, +++ moderate, ++++ marked inflammatory reaction; *no statistically significant difference (*P* = 0.19).
